# 2-Hy­droxy­methyl-1,3-dimethyl-1*H*-benzimidazol-3-ium iodide

**DOI:** 10.1107/S1600536813022307

**Published:** 2013-08-14

**Authors:** Mohamed El Hadi Said, Sofiane Bouacida, Hocine Merazig, Ali Belfaitah, Aissa Chibani, Abdelmalek Bouraiou

**Affiliations:** a Unité de Recherche de Chimie de l’Environnement et Moléculaire Structurale, CHEMS, Université Constantine 1, 25000, Algeria; b Laboratoire des Produits Naturels d’Origine Végétale et de Synthèse Organique, PHYSYNOR, Université Constantine 1, 25000 Constantine, Algeria

## Abstract

In the cation of the title compound, C_10_H_13_N_2_O^+^·I^−^, all non-H atoms, with the exception of the O atom, are essentially coplanar, with a maximum deviation of 0.04 (1) Å. In the crystal, the cations and anions are arranged in layers parallel to (100). The cations are connected to the anions *via* an O—H⋯I hydrogen bond and there are significant π–π stacking inter­actions between cation layers, with centroid–centroid distances in the range 3.606 (5)–3.630 (5) Å. A weak intra­molecular C—H⋯O hydrogen bond is also observed. The crystal studied was an inversion twin with refined components of 0.52 (5) and 0.48 (5).

## Related literature

For applications of this class of compounds, see: Tonelli *et al.* (2010 ▶); Preston (1974 ▶); Hazelton *et al.* (1995 ▶); Kucukguzel *et al.* (2001 ▶); Islam *et al.* (1991 ▶); Li *et al.* (2003 ▶); Abboud *et al.* (2006 ▶). For our previous work on imidazole derivatives, see: Bahnous *et al.* (2012 ▶); Zama *et al.* (2013 ▶); Chelghoum *et al.* (2011 ▶).
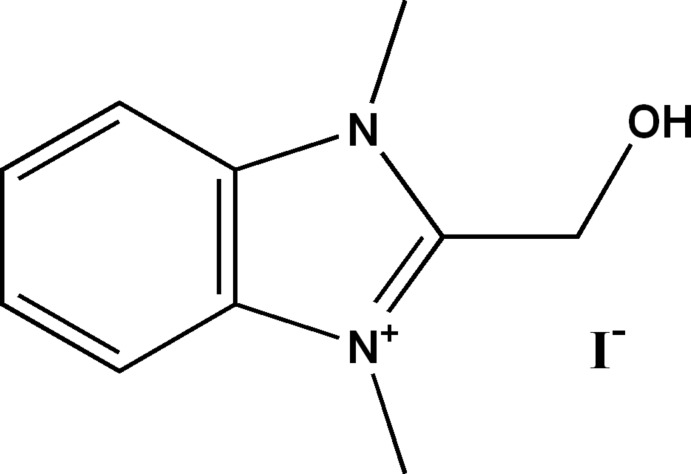


## Experimental

### 

#### Crystal data

C_10_H_13_N_2_O^+^·I^−^
*M* *_r_* = 304.12
Orthorhombic, 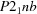

*a* = 6.5690 (7) Å
*b* = 10.1342 (10) Å
*c* = 16.9357 (19) Å
*V* = 1127.4 (2) Å^3^
*Z* = 4
Mo *K*α radiation
μ = 2.81 mm^−1^
*T* = 150 K
0.14 × 0.13 × 0.12 mm

#### Data collection

Bruker APEXII CCD diffractometer
Absorption correction: multi-scan (*SADABS*; Sheldrick, 2002 ▶) *T*_min_ = 0.647, *T*_max_ = 0.747
10018 measured reflections
4002 independent reflections
3243 reflections with *I* > 2σ(*I*)
*R*_int_ = 0.02

#### Refinement

*R*[*F* ^2^ > 2σ(*F* ^2^)] = 0.027
*wR*(*F* ^2^) = 0.068
*S* = 1.05
4002 reflections
131 parameters
1 restraint
H-atom parameters constrained
Δρ_max_ = 1.34 e Å^−3^
Δρ_min_ = −0.72 e Å^−3^
Absolute structure: Flack (1983 ▶), 1518 Friedel pairs
Absolute structure parameter: 0.48 (5)

### 

Data collection: *APEX2* (Bruker, 2006 ▶); cell refinement: *SAINT* (Bruker, 2006 ▶); data reduction: *SAINT*; program(s) used to solve structure: *SIR2002* (Burla *et al.*, 2005 ▶); program(s) used to refine structure: *SHELXL97* (Sheldrick, 2008 ▶); molecular graphics: *ORTEP-3 for Windows* (Farrugia, 2012 ▶) and *DIAMOND* (Brandenburg & Berndt, 2001 ▶); software used to prepare material for publication: *WinGX* (Farrugia, 2012 ▶) and *CRYSCAL* (T. Roisnel, local program).

## Supplementary Material

Crystal structure: contains datablock(s) I. DOI: 10.1107/S1600536813022307/lh5638sup1.cif

Structure factors: contains datablock(s) I. DOI: 10.1107/S1600536813022307/lh5638Isup2.hkl

Supplementary material file. DOI: 10.1107/S1600536813022307/lh5638Isup3.cml

Additional supplementary materials:  crystallographic information; 3D view; checkCIF report

## Figures and Tables

**Table 1:** Hydrogen-bond geometry (Å, °)

*D*—H⋯*A*	*D*—H	H⋯*A*	*D*⋯*A*	*D*—H⋯*A*
O1—H1⋯I1^i^	0.82	2.66	3.473 (3)	171
C5—H5*A*⋯O1	0.96	2.52	3.170 (5)	125

Symmetry code: (i) 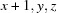.

**Fig. 1.**
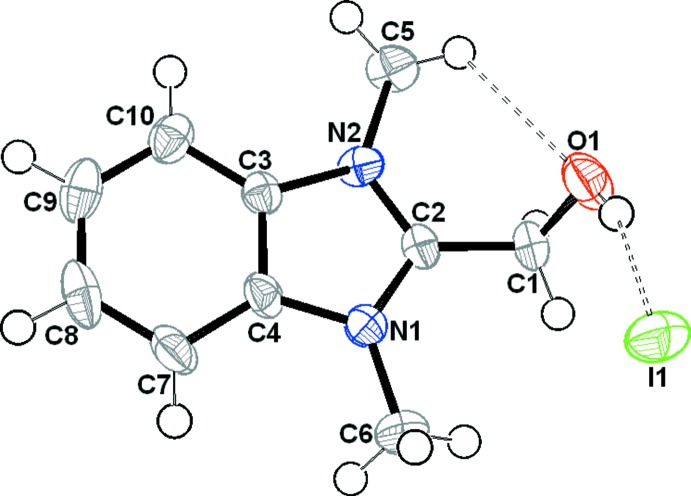
The molecular structure of (I) with displacement ellipsoids drawn at the 50% probability level. H atoms are represented as small spheres of arbitrary radius. Hydrogen bonds are shown as dashed lines.

**Fig. 2.**
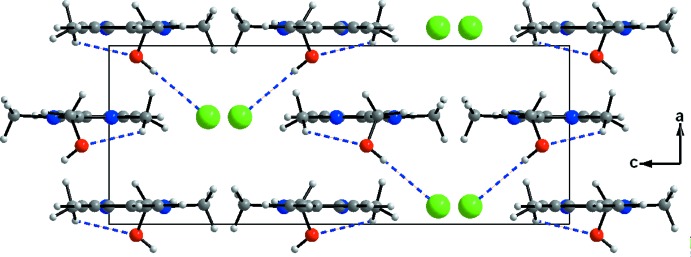
Alternating layers of (I) viewed along the b axis showing hydrogen bonds as dashed lines.

## supplementary crystallographic information

### 1. Comment

Benzimidazole, is isosteric with indole and purine nuclei, which are present in a number of fundamental cellular components and bioactive compounds. This structural similarity means the benzimidazole molecule is endowed with a variety of interesting biological properties (Kucukguzel, *et al.*, 2001; Islam *et al.*, 1991; Tonelli *et al.*, 2010). Some of these compounds are marketed as antifungal, (Preston, 1974), antihelmintic, (Hazelton *et al.*, 1995). Furthermore, benzimidazole derivatives can be also used as epoxy resin curing agents, catalysts, and metallic surface treatment agents (Li *et al.*, 2003; Abboud *et al.*, 2006). In previous work, we have reported the synthesis and structure determination of some new heterocyclic compounds bearing an imidazole unit (Bahnous *et al.*, 2012; Zama *et al.*, 2013; Chelghoum *et al.*, 2011). Herein, we describe the synthesis and the structure determination of 1,3-dimethyl-2-hydroxymethylbenzimidazolium iodide (I) resulting from the quaternization reaction of 1-methyl-2-hydroxymethylbenzimidazole with methyl iodide.

The molecular structure of (I) is shown in Fig. 1. The asymmetric unit contains a 1,3-dimethyl-2-hydroxymethylbenzimidazolium cation and an iodide anion. All non-H atoms in the cation, with the exception of the O atom, are essentially co-planar with a maximum deviation of 0.04 (1)Å for N1. In the crystal, the cations and anions are arranged in layers parallel to (100) (Fig. 2). The cations are hydrogen bonded to the anions via an O—H···I hydrogen bond and there are significant π—π stacking interactions between cation layers with centroid-centroid distances in the range 3.606 (5) - 3.630 (5)Å.

### 2. Experimental

To a solution of 1-methyl-2-hydroxymethylbenzimidazole derivatives (10 mmol) in 20 ml of acetonitrile, was added 30 mmol of methyl iodide. The reaction mixture was refluxed. When the reaction was over (TLC), the solvent volume was reduced and the crude product was then filtered off and washed with cold acetonitrile. Suitable crystals for X-ray analysis were obtained by slow evaporation of a water solution of (I).

### 3. Refinement

Approximate positions for all H atoms were first obtained from the difference electron density map. However, the H atoms were ultimately placed in idealized positions and treated as riding. The applied constraints were as follows: C_aryl_—H_aryl_ = 0.93 Å; C_methylene_—H_methylene_ = 0.97 Å; C_methyl_—H_methyl_ = 0.96 Å and C_hydroxy_—H_hydroxy_ = 0.82 Å. The idealized methyl group was allowed to rotate about the C—C bond during the refinement by application of the command AFIX 137 in *SHELXL97* (Sheldrick, 2008). *U*_iso_(H_methyl_ or H_hydroxy_) = 1.5*U*_eq_(C_methyl_ or O_hydroxy_) or *U*_iso_(H_aryl_ or H_methylene_) = 1.2 *U*_eq_(C_aryl_ or C_methylene_). The crystal used is an inversion twin with refined components 0.52 (5) and 0.48 (5).

### Crystal data

**Table:**

C_10_H_13_N_2_O^+^·I^−^	*F*(000) = 592
*M**_r_* = 304.12	*D*_x_ = 1.792 Mg m^−^^3^
Orthorhombic, *P*2_1_*n**b*	Mo *K*α radiation, λ = 0.71073 Å
Hall symbol: P -2bc 2a	Cell parameters from 4274 reflections
*a* = 6.5690 (7) Å	θ = 2.4–34.0°
*b* = 10.1342 (10) Å	µ = 2.81 mm^−^^1^
*c* = 16.9357 (19) Å	*T* = 150 K
*V* = 1127.4 (2) Å^3^	Cube, colorless
*Z* = 4	0.14 × 0.13 × 0.12 mm

### Data collection

**Table:**

Bruker APEXII CCD diffractometer	3243 reflections with *I* > 2σ(*I*)
Graphite monochromator	*R*_int_ = 0.02
CCD rotation images, thin slices scans	θ_max_ = 34.2°, θ_min_ = 3.1°
Absorption correction: multi-scan (*SADABS*; Sheldrick, 2002)	*h* = −9→10
*T*_min_ = 0.647, *T*_max_ = 0.747	*k* = −16→15
10018 measured reflections	*l* = −26→19
4002 independent reflections	

### Refinement

**Table:**

Refinement on *F*^2^	Secondary atom site location: difference Fourier map
Least-squares matrix: full	Hydrogen site location: inferred from neighbouring sites
*R*[*F*^2^ > 2σ(*F*^2^)] = 0.027	H-atom parameters constrained
*wR*(*F*^2^) = 0.068	*w* = 1/[σ^2^(*F*_o_^2^) + (0.0263*P*)^2^ + 1.1204*P*] where *P* = (*F*_o_^2^ + 2*F*_c_^2^)/3
*S* = 1.05	(Δ/σ)_max_ = 0.003
4002 reflections	Δρ_max_ = 1.34 e Å^−^^3^
131 parameters	Δρ_min_ = −0.72 e Å^−^^3^
1 restraint	Absolute structure: Flack (1983), 1518 Friedel pairs
Primary atom site location: structure-invariant direct methods	Absolute structure parameter: 0.48 (5)

### Special details

**Table:**

Geometry. Bond distances, angles *etc*. have been calculated using the rounded fractional coordinates. All su's are estimated from the variances of the (full) variance-covariance matrix. The cell e.s.d.'s are taken into account in the estimation of distances, angles and torsion angles
Refinement. Refinement of *F*^2^ against ALL reflections. The weighted *R*-factor *wR* and goodness of fit *S* are based on *F*^2^, conventional *R*-factors *R* are based on *F*, with *F* set to zero for negative *F*^2^. The threshold expression of *F*^2^ > σ(*F*^2^) is used only for calculating *R*-factors(gt) *etc*. and is not relevant to the choice of reflections for refinement. *R*-factors based on *F*^2^ are statistically about twice as large as those based on *F*, and *R*- factors based on ALL data will be even larger.

### Fractional atomic coordinates and isotropic or equivalent isotropic displacement parameters (Å^2^)

**Table:**

	*x*	*y*	*z*	*U*_iso_*/*U*_eq_	
O1	1.0638 (4)	0.0386 (3)	0.05918 (17)	0.0303 (8)	
N1	0.9055 (15)	0.3459 (2)	0.11990 (13)	0.0203 (5)	
N2	0.9033 (12)	0.2982 (2)	−0.00606 (12)	0.0168 (5)	
C1	0.893 (2)	0.1018 (3)	0.0837 (2)	0.0284 (9)	
C2	0.9025 (18)	0.2473 (2)	0.06700 (14)	0.0192 (5)	
C3	0.9014 (18)	0.4343 (2)	−0.00046 (14)	0.0165 (5)	
C4	0.9022 (15)	0.4649 (2)	0.07953 (15)	0.0179 (6)	
C5	0.9046 (16)	0.2214 (3)	−0.07961 (15)	0.0226 (7)	
C6	0.893 (2)	0.3340 (3)	0.20631 (18)	0.0326 (11)	
C7	0.901 (2)	0.5947 (2)	0.10657 (18)	0.0239 (6)	
C8	0.9001 (18)	0.6923 (2)	0.0495 (2)	0.0277 (7)	
C9	0.9004 (16)	0.6620 (3)	−0.03096 (19)	0.0255 (7)	
C10	0.9028 (14)	0.5327 (2)	−0.05820 (16)	0.0218 (6)	
I1	0.3999 (3)	0.08238 (2)	0.21526 (1)	0.0330 (1)	
H1	1.15370	0.04750	0.09236	0.0454*	
H1A	0.77590	0.06427	0.05712	0.0341*	
H1B	0.87606	0.08812	0.13999	0.0341*	
H5A	0.98363	0.14266	−0.07223	0.0339*	
H5B	0.76764	0.19799	−0.09352	0.0339*	
H5C	0.96352	0.27334	−0.12114	0.0339*	
H6A	0.83795	0.24932	0.21993	0.0487*	
H6B	1.02677	0.34281	0.22861	0.0487*	
H6C	0.80648	0.40223	0.22679	0.0487*	
H7	0.90136	0.61465	0.16017	0.0287*	
H8	0.89907	0.78024	0.06512	0.0333*	
H9	0.89902	0.73060	−0.06745	0.0306*	
H10	0.90517	0.51273	−0.11180	0.0262*	

### Atomic displacement parameters (Å^2^)

**Table:**

	*U*^11^	*U*^22^	*U*^33^	*U*^12^	*U*^13^	*U*^23^
O1	0.0335 (14)	0.0250 (11)	0.0324 (14)	0.0107 (11)	−0.0066 (12)	−0.0073 (10)
N1	0.0282 (11)	0.0161 (8)	0.0166 (9)	−0.002 (3)	0.000 (4)	−0.0013 (7)
N2	0.0179 (9)	0.0168 (8)	0.0158 (9)	−0.011 (2)	0.001 (3)	−0.0022 (7)
C1	0.045 (2)	0.0144 (10)	0.0258 (13)	0.009 (3)	0.004 (4)	0.0015 (9)
C2	0.0215 (10)	0.0150 (8)	0.0210 (10)	0.003 (3)	0.003 (5)	−0.0004 (8)
C3	0.0142 (8)	0.0155 (9)	0.0197 (10)	−0.001 (3)	0.001 (5)	−0.0004 (7)
C4	0.0179 (10)	0.0152 (9)	0.0206 (11)	0.006 (3)	−0.003 (4)	−0.0020 (8)
C5	0.0248 (13)	0.0236 (11)	0.0194 (11)	−0.010 (3)	−0.003 (4)	−0.0061 (9)
C6	0.053 (3)	0.0289 (12)	0.0158 (12)	0.003 (4)	−0.001 (4)	−0.0010 (9)
C7	0.0250 (11)	0.0174 (9)	0.0292 (13)	0.005 (4)	0.001 (6)	−0.0065 (9)
C8	0.0222 (11)	0.0130 (9)	0.0480 (17)	0.000 (4)	−0.001 (6)	−0.0015 (10)
C9	0.0183 (11)	0.0205 (10)	0.0377 (15)	−0.008 (3)	−0.003 (5)	0.0089 (10)
C10	0.0188 (10)	0.0226 (10)	0.0240 (12)	0.002 (4)	−0.001 (5)	0.0059 (9)
I1	0.0493 (1)	0.0319 (1)	0.0177 (1)	−0.0001 (3)	−0.0001 (4)	−0.0009 (1)

### Geometric parameters (Å, º)

**Table:**

O1—C1	1.357 (11)	C9—C10	1.389 (4)
O1—H1	0.8200	C1—H1A	0.9700
N1—C4	1.387 (3)	C1—H1B	0.9700
N1—C6	1.471 (4)	C5—H5A	0.9600
N1—C2	1.342 (3)	C5—H5B	0.9600
N2—C3	1.383 (3)	C5—H5C	0.9600
N2—C5	1.469 (3)	C6—H6A	0.9600
N2—C2	1.341 (3)	C6—H6B	0.9600
C1—C2	1.503 (4)	C6—H6C	0.9600
C3—C10	1.397 (3)	C7—H7	0.9300
C3—C4	1.390 (3)	C8—H8	0.9300
C4—C7	1.393 (3)	C9—H9	0.9300
C7—C8	1.383 (4)	C10—H10	0.9300
C8—C9	1.397 (5)		
			
C1—O1—H1	109.00	C2—C1—H1A	109.00
C2—N1—C4	108.6 (2)	C2—C1—H1B	109.00
C2—N1—C6	127.0 (2)	H1A—C1—H1B	108.00
C4—N1—C6	124.1 (2)	N2—C5—H5A	109.00
C2—N2—C3	108.69 (19)	N2—C5—H5B	109.00
C2—N2—C5	125.4 (2)	N2—C5—H5C	109.00
C3—N2—C5	125.9 (2)	H5A—C5—H5B	109.00
O1—C1—C2	111.8 (8)	H5A—C5—H5C	109.00
N1—C2—C1	127.3 (2)	H5B—C5—H5C	109.00
N2—C2—C1	123.5 (2)	N1—C6—H6A	109.00
N1—C2—N2	109.24 (19)	N1—C6—H6B	109.00
N2—C3—C10	131.6 (2)	N1—C6—H6C	109.00
C4—C3—C10	121.5 (2)	H6A—C6—H6B	109.00
N2—C3—C4	106.82 (19)	H6A—C6—H6C	110.00
N1—C4—C7	131.3 (2)	H6B—C6—H6C	109.00
C3—C4—C7	122.1 (2)	C4—C7—H7	122.00
N1—C4—C3	106.66 (19)	C8—C7—H7	122.00
C4—C7—C8	116.5 (3)	C7—C8—H8	119.00
C7—C8—C9	121.6 (2)	C9—C8—H8	119.00
C8—C9—C10	122.1 (3)	C8—C9—H9	119.00
C3—C10—C9	116.2 (2)	C10—C9—H9	119.00
O1—C1—H1A	109.00	C3—C10—H10	122.00
O1—C1—H1B	109.00	C9—C10—H10	122.00
			
C2—N1—C4—C7	179.2 (12)	C3—N2—C2—C1	176.7 (11)
C6—N1—C4—C7	4.8 (19)	O1—C1—C2—N2	66.5 (13)
C4—N1—C2—N2	1.8 (13)	O1—C1—C2—N1	−115.6 (11)
C6—N1—C2—N2	175.9 (10)	N2—C3—C10—C9	−179.9 (11)
C4—N1—C2—C1	−176.4 (11)	C4—C3—C10—C9	−1.4 (15)
C6—N1—C2—C1	−2 (2)	C10—C3—C4—C7	1.1 (17)
C6—N1—C4—C3	−175.6 (10)	N2—C3—C4—N1	0.3 (12)
C2—N1—C4—C3	−1.2 (12)	N2—C3—C4—C7	179.9 (10)
C5—N2—C2—N1	178.8 (9)	C10—C3—C4—N1	−178.6 (10)
C5—N2—C2—C1	−3.0 (17)	N1—C4—C7—C8	179.2 (11)
C3—N2—C2—N1	−1.6 (13)	C3—C4—C7—C8	−0.4 (17)
C5—N2—C3—C4	−179.6 (9)	C4—C7—C8—C9	0.0 (18)
C2—N2—C3—C10	179.5 (12)	C7—C8—C9—C10	−0.4 (18)
C2—N2—C3—C4	0.8 (12)	C8—C9—C10—C3	1.1 (15)
C5—N2—C3—C10	−0.9 (18)		

### Hydrogen-bond geometry (Å, º)

**Table:**

*D*—H···*A*	*D*—H	H···*A*	*D*···*A*	*D*—H···*A*
O1—H1···I1^i^	0.8200	2.6600	3.473 (3)	171.00
C5—H5*A*···O1	0.9600	2.5200	3.170 (5)	125.00

Symmetry code: (i) *x*+1, *y*, *z*.
